# Fracture Toughness Analysis of Ni–Cr–Mo Low-Alloy Steel for Advanced Nuclear Power

**DOI:** 10.3390/ma18163743

**Published:** 2025-08-11

**Authors:** Xiaochuan Zeng, Yili Huang, Mingjie Guo, Cuizhu He, Qiaodan Hu

**Affiliations:** 1 China Nuclear Power Engineering Co., Ltd., Shenzhen 518116, China; xie25@126.com (X.Z.); hyl3690@126.com (Y.H.); leoncook@163.com (M.G.); xinyu11.love@163.com (C.H.); 2 Institution of Advanced Materials and Solidification, Shanghai Jiao Tong University, Shanghai 200240, China

**Keywords:** Cr–Ni–Mo low-alloy steel, fracture toughness, microstructure

## Abstract

The fracture toughness of nuclear reactor pressure vessel (RPV) steel is an important basis for the structural integrity evaluation of equipment. SA508 Gr.4N (Cr–Ni–Mo) low-alloy steel has attracted people’s attention because of its excellent strength and toughness, and it is considered as a candidate material for the next generation of RPV. The fracture toughness of SA508 Gr.4N alloy steel was analyzed from the perspective of macroscopic mechanical properties and microstructure, and compared with that of the SA508 Gr.3 (Mn–Ni–Mo) steel used in commercial PWR nuclear power plants. SA508 Gr.4N steel showed better toughness reserve than SA508 Gr.3 steel in terms of fracture toughness parameters such as the reference nil-ductility transition temperature RT_NDT_, brittleness transition characteristic temperature T_41J_, upper shelf energy and master curve reference temperature T_0_. The reasons for the excellent fracture toughness of SA508 Gr.4N steel were analyzed from the aspects of microstructure, precipitation and grain boundary structure.

## 1. Introduction

At present, Mn–Ni–Mo alloy steel (such as American SA533 Gr.B steel, SA508 Gr.3 steel and French 16MND5 steel) is the mainstream material for reactor pressure vessel (RPV) pressure-retaining boundaries of PWR nuclear power plants (NPPs). It has been used in NPPs for 40 years because of its good mechanical properties, weldability and certain economic benefits [1,2]. With the development of NPPs towards high power, large scale and long life, traditional Mn–Ni–Mo alloy steels used in RPV are facing challenges including insufficient hardenability, inadequate radiation resistance, fracture toughness nearing their limits and insufficient safety margins [3,4,5]. In order to solve the problems of poor hardenability, inhomogeneity microstructure across the thickness and unstable mechanical properties of large-sized Mn–Ni–Mo alloy forgings, on the basis of SA508 Gr.3 steel, ASTM has developed SA508 Gr.4N steel (Ni–Cr–Mo steel) with better hardenability and toughness by increasing Ni and Cr content and reducing Mn content to eliminate the possible segregation [6,7]. It is expected to be the main RPV material for NPPs in the future.

In the design and manufacture of RPVs, the fracture toughness of materials is a crucial performance index, and the toughness loss of RPV steel caused by irradiation embrittlement [8] is one of the key mechanisms of equipment failure. Therefore, it is very important to study the fracture toughness of RPV steel for the safe operation and life management of NPPs. Through adjusting the content of Ni, Cr and Mn elements, SA508 Gr.4N steel has optimized the microstructure and precipitation of the second phase. These optimizations can significantly improve the high-temperature strength and environmental embrittlement resistance [9]. Nevertheless, the fracture toughness characteristics of SA508 Gr.4N steel in the ductile–brittle transition temperature (DBTT) zone have not been clearly defined, and the relationship between the fracture mechanism and microstructure evolution still needs further exploration [10]. Existing studies have shown that the fracture toughness of SA508 steel is closely related to its microstructure. For example, the low-temperature impact toughness of a ferrite + bainite structure may decrease due to the high nucleation tendency of grain boundary cracks, while the martensite lath can significantly improve toughness because its high-density and large-angle interfaces can inhibit crack propagation [11]. The microstructure of SA508 Gr.4N steel usually contains bainite, martensite and residual austenite. The complex phase transition behavior may further affect the fracture path [12,13]. Existing research mainly focuses on the effects of the solute elements or metallography composition on the fracture toughness and tempering embrittlement of SA508 Gr.4N steel. But the fracture mechanism of SA508 Gr.4N steel in the ductile–brittle transition zone is still not clear. The microscopic mechanism of crack propagation in the DBTT region, such as the secondary cracking behavior and the competition between cleavage fracture and dimple fracture, has not been fully revealed.

The purpose of this study is to comprehensively analyze the fracture properties of SA508 Gr.4N steel through tensile properties, impact fracture and fracture toughness in the ductile–brittle transition zone. The characteristics of fracture toughness in the ductile–brittle transition zone were investigated from the metallographic structure, fracture scanning and electron backscattering diffraction (EBSD) technology of fracture specimens. The abovementioned macroscopic and microscopic properties of SA508 Gr.4N steel were also compared with those of traditional SA508 Gr.3 steel. Through these comprehensive analyses, we expect to achieve a more comprehensive understanding of the fracture toughness mechanism of SA508 Gr.4N steel. Therefore, it can provide a scientific basis for the property optimization and reliability evaluation of this material in critical applications such as RPVs. In addition, the findings of this study will also provide an important reference and guidance for the development of a new generation of low-alloy steels with better fracture toughness.

## 2. Materials and Methods

### 2.1. Test Materials

The SA508 Gr.4N steel and SA508 Gr.3 steel used in the test were obtained from industrial (commercial) forgings. In accordance with ASME specifications, the forgings were made by double vacuum smelting. Molten steel was produced in an electric furnace and refined in a refining furnace with aluminum killing and vacuum degassing. The vacuum pouring process, which allows precise control of the chemical composition, reducing the content of harmful elements and inclusions in molten steel, was used in the steel making process. The results of the heat analysis of both materials are shown in Table 1.

The forging reduction ratio of both materials was greater than 3. The heat treatment process of the material after forging was as follows: SA508 Gr.4N steel was quenched at 880 °C for 3.5 h, cooled in flowing water, and then tempered at 650 °C for about 20 h. The quenching temperature of SA508 Gr.3 steel was 880 °C, held for 4 h, and then cooled in flowing water. Subsequently, SA508 Gr.3 steel was tempered at 650 °C for about 20 h. When tempering, the rising and cooling rate above 350 °C did not exceed 150 °C/h. Due to the fact that SA508 Gr.4N steel has better hardenability and a larger austenite range caused by the Ni element, the quenching time of SA508 Gr.4N steel was slightly shorter than that of SA508 Gr.3 steel.

**Figure 1:**
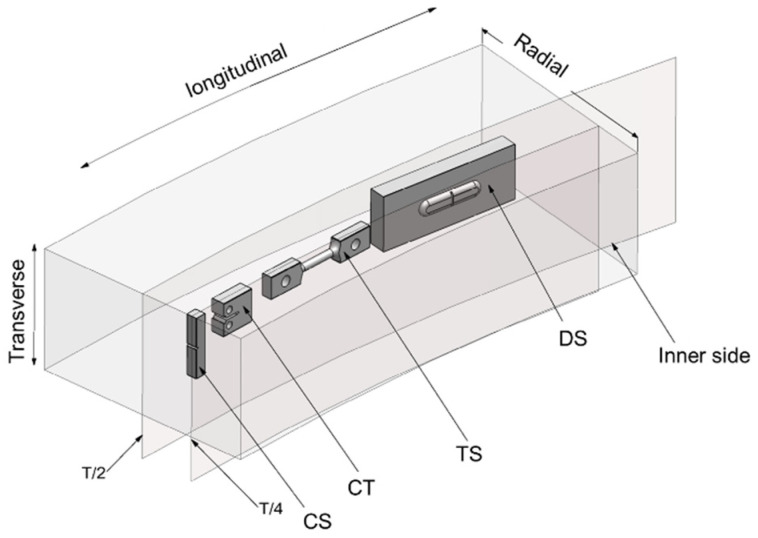
Schematic diagram of specimens’ direction and location. T is the thickness of the forging. CS represents the Cv impact specimen, CT represents the fracture toughness specimen, TS represents tensile specimen, and DS represents the Pellini drop weight specimen.

All the samples were cut from the 1/4 thickness position of the forgings. The tensile specimens were in the longitudinal direction, the Charpy-V type (Cv) impact specimens were in the transverse direction, the Pellini drop weight specimens were in the longitudinal direction, and the crack growth direction of the fracture toughness specimens was in the longitudinal direction of the forgings. All specimen types were taken as shown in Figure 1.

### 2.2. Test Methods

The tensile tests at room temperature and Cv impact tests were performed in accordance with ASTM A370-19 [14], and the tensile tests at 290 °C were performed in accordance with ASTM E21-20 [15]. The tensile test specimens were Ø6.35 mm sized. The sample size of 10 mm × 10 mm × 55 mm was used for Cv impact testing.

The reference nil-ductility transition temperature (RT_NDT_) of the material was determined by both the non-ductile transition temperature (NDTT) and the Cv impact test. Firstly, the NDTT was determined from the Pellini drop weight test according to the method specified in ASTM E208-20 [16]. The Pellini drop weight test specimens were of the size type P-3. Secondly, the Cv impact tests were started on the basis of NDTT + 33 °C, and every three specimens were used as a group. When the E(Cv) at this temperature was ≥68 J and the lateral expansion was ≥0.89 mm, RT_NDT_ = NDTT.

**Figure 2:**
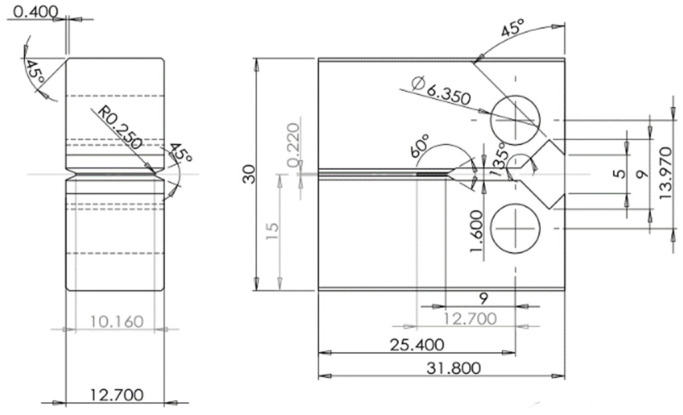
CT specimen for low-temperature fracture toughness test.

The fracture toughness during the ductile–brittle transition zone was tested according to the single temperature method specified in ASTM E1921-19b [17]. The compact tension (CT) specimen was selected, the thickness of the specimen was 12.7 mm, and the specific dimensions are shown in Figure 2. There was a side groove on each side of the specimen. The stress intensity factor K was used to control the preformed fatigue crack, and the maximum stress intensity factor K_max_ was gradually reduced by continuous smoothing. Fatigue pre-cracking was conducted at room temperature with a stress ratio (R) of 0.1. The test temperature was preset according to ASTM E1921-19b [17]. The temperature of the test specimen was controlled by the low-temperature environment chamber, in which the temperature sensor did not have contact with the specimen. The cooling medium during the test was liquid nitrogen. The self-pressurized liquid nitrogen tank and solenoid valve were used to realize automatic temperature control, and the temperature control error was less than ±0.3 °C. After reaching the preset temperature, the specimen was maintained for at least 30 min to ensure temperature homogeneity and then the test was started.

The microstructure of fracture characteristics was characterized by an optical microscopy (OM) (Axio Scope A1, Zeiss, Köln, Germany) and a scanning electron microscopy (SEM) (Phenom Pro, Eindhoven, The Netherlands). The specimens for the OM were corroded by 4% nitric acid solution and then air dried in a drying oven.

The specimens examined near the ductile and brittle transition temperature were selected for fracture morphology analysis. The grain orientation and grain boundary distribution were analyzed using electron backscattering diffraction (EBSD). The carbide precipitates distributed at the grain boundaries and within grains were analyzed using the electron energy spectrum (EDS).

## 3. Results

### 3.1. Tensile Properties

After heat treatment specified in Section 2.1, tensile tests were carried out at room temperature and 290 °C, which represented the mechanical strength of the material at room temperature and service temperature, respectively. The tensile test results are shown in Table 2. The results of all specimens complied with the ASME code requirements. The yield strength and tensile strength of SA508 Gr.4N steel were about 50% and 40% higher than those of SA508 Gr.3 steel, respectively, at both room and high temperatures. However, the elongation and section shrinkage of SA508 Gr.4N steel were about 10% lower than those of SA508 Gr.3 steel. SA508 Gr.4N steel exhibited much higher strength than SA508 Gr.3 steel.

### 3.2. Cv Impact Toughness and RT_NDT_

**Figure 3:**
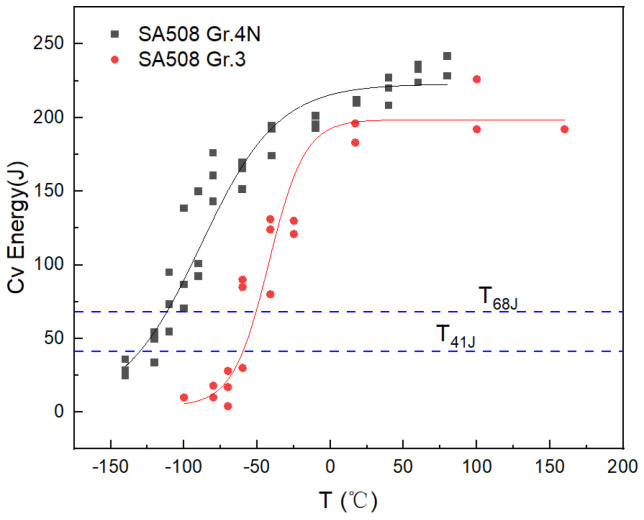
Comparison of Cv impact curves between SA508 Gr.4N steel and SA508 Gr.3 steel.

The Cv impact tests at a series of temperatures were carried out, and the results are shown in Figure 3. The black square and the red dot are the Cv impact energy of SA508 Gr.4N steel and SA508 Gr.3 steel, respectively. The impact transition curve (black line and red line) was fitted according to the hyperbolic tangent function. The characteristic values of the Cv impact test and RT_NDT_ results are shown in Table 3. The upper shelf energy (USE) of SA508 Gr.4N steel was 24.3 J higher than that of SA508 Gr.3 steel, while the characteristic temperatures T_41J_ and T_68J_ of SA508 Gr.4N steel were 70 °C and 61.2 °C lower than those of SA508 Gr.3 steel, respectively. With the content of Cr and Ni elements increased, SA508 Gr.4N steel showed better toughness in the low-temperature region than SA508 Gr.3 steel. Moreover, the RT_NDT_ of SA508 Gr.4N steel was approximately 90 °C lower than that of SA508 Gr.3 steel, which was a significant improvement compared to the −12 °C required in the ASME code [2]. As for the fracture toughness index (RT_NDT_) used in engineering evaluation, SA508 Gr.4N steel performed particularly well.

### 3.3. Fracture Toughness

The fracture toughness of SA508 Gr.4N steel and SA508 Gr.3 steel was tested using CT specimens with side grooves according to ASTM E1921-19b [17]. The relationship between the fracture toughness K_JC_ with a cumulative failure probability of P_f_ and temperature is as follows:

(1) $$K_{JC(P_{f})}=K_{min}+{\left[ln\left(\frac{1}{1-P_{f}}\right)\right]}^{1/4}\left\{11+77exp\left[0.019\left(T-T_{0}\right)\right]\right\}$$

where K_min_ is assumed to be 20 MPa m^0.5^, P_f_ is the cumulative failure probability, T is the test temperature and T_0_ is the reference temperature.

The expression of the master curve K_JC(med)_ corresponding to the cumulative failure probability of 50% is as follows:

(2) $$K_{JC(med)}=30+70exp\left[0.019\left(T-T_{0}\right)\right]$$

**Figure 4:**
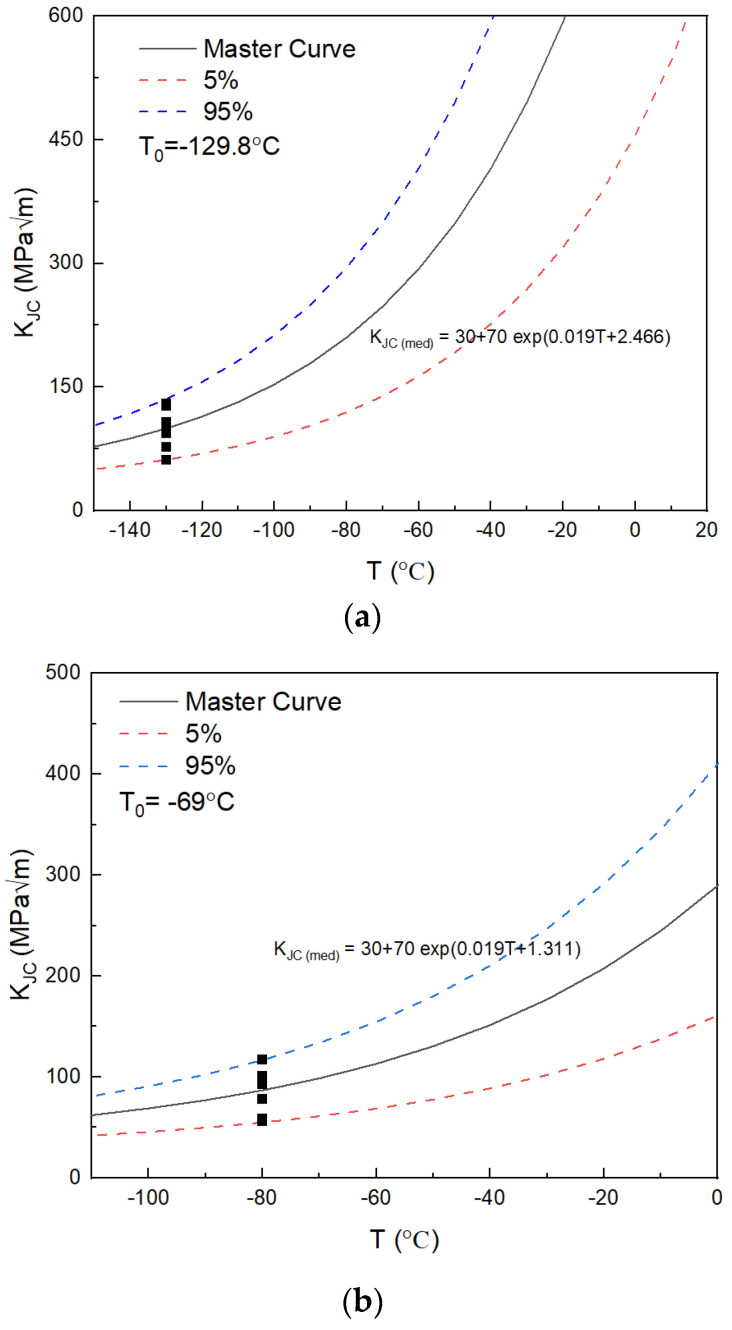
Standard master curve and K_JC_ value for (**a**) SA508 Gr.4N steel and (**b**) SA508 Gr.3 steel. The black boxes are the values of K_JC_.

The master curve characterization of the fracture toughness for the two materials is shown in Figure 4. The black solid line is the median line of the master curve, representing a 50% failure probability. The red and blue dashed lines represent the cumulative failure probability curves of 5% and 95%, respectively. The measured K_JC_ was converted to the equivalent 1T specimen. The discreteness of fracture toughness data for SA508 Gr.3 steel can be well explained by the master curve and tolerance boundary. The temperature dependence of fracture toughness for SA508 Gr.4N steel was greater than that predicted by the standard master curve specified in the ASTM E1921-19b [17]. According to the conclusions of references [18,19], this may be related to the tempered martensitic structure of SA508 Gr.4N.

The reference temperature T_0_ of the fracture toughness for SA508 Gr.4N steel in the ductile brittle transition zone was −129.8 °C, and the reference temperature T_0_ of SA508 Gr.3 steel was −69 °C. The T_0_ of SA508 Gr.4N steel is 60 °C lower than that of SA508 Gr.3 steel, which is consistent with the results of Kim’s research [9].

### 3.4. Microstructure Analysis

#### 3.4.1. Optical Microscope

**Figure 5:**
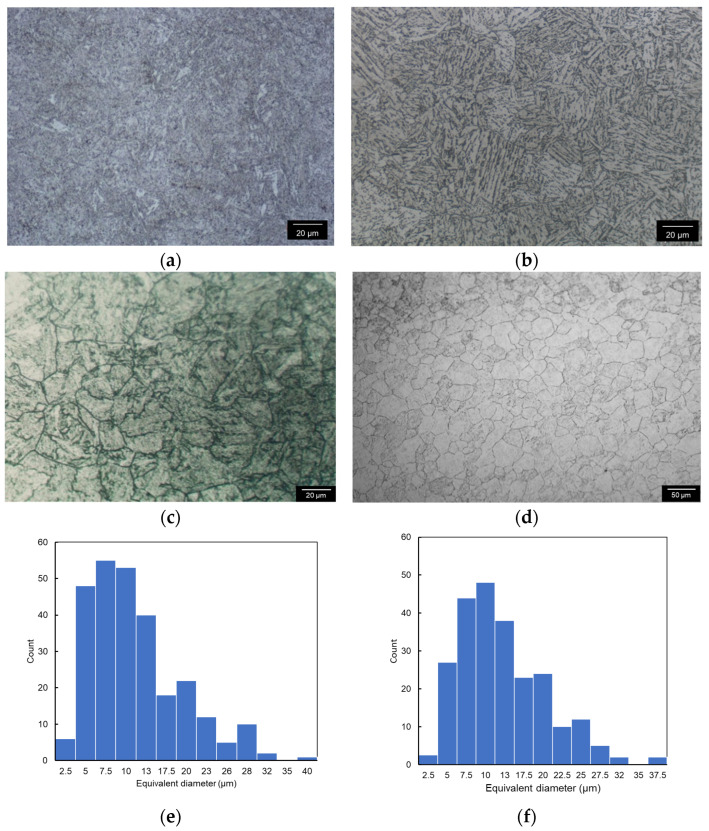
Optical microscope photographs of SA508 Gr.4N steel and SA508 Gr.3 steel: (**a**,**b**) represent the optical microstructure of SA508 Gr.4N steel and SA508 Gr.3 steel, (**c**,**d**) represent the grain size of SA508 Gr.4N steel and SA508 Gr.3 steel, (**e**,**f**) represent the grain size statistics of SA508 Gr.4N steel and SA508 Gr.3 steel.

The structure photos and grain size diagrams of SA508 Gr.4N steel and SA508 Gr.3 steel from the optical microscope are shown in Figure 5. The optical microstructure of the two materials in the picture is relatively complex. The mixed structure of martensite lath (ML) and a small amount of bainite lath (BL) was formed in the SA508 Gr.4N steel, while the bainite was mainly formed in the SA508 Gr.3 steel sample. The microstructure size of SA508 Gr.4N steel was smaller than that of SA508 Gr.3 steel. In the microstructure of the two materials, a large number of dispersed carbide particles can be observed, which is due to the diffusion of carbon atoms and alloy atoms during the tempering process, and the carbide can be fully precipitated. According to the scanning electron microscopy (SEM) analyses in Section 3.4.2 and reference [19], most of these fine precipitates were likely to be Cr-rich M7C3, M23C6 (fcc) carbides and a small amount of Mo-rich M2C (compact hexagonal structure).

The sample used for grain size was treated with supersaturated picric acid, and a clear twin structure in SA508 Gr.4N steel could be observed. The average grain diameter of SA508 Gr.4N steel is about 11 μm, and the grain size number is 6.5, which is similar to SA508 Gr.3 steel with an average grain diameter of about 10 μm and grain size number of 7.

#### 3.4.2. Scanning Electron Microscope

**Figure 6:**
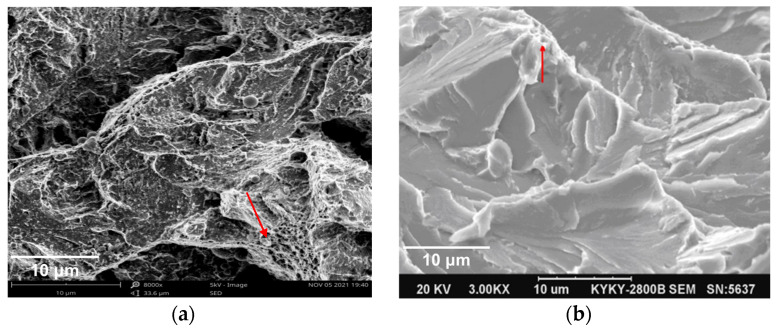
SEM images of the fracture morphology of (**a**) SA508 Gr.4N steel and (**b**) SA508 Gr.3 steel CT samples.

The fracture morphology of SA508 Gr.4N steel and SA508 Gr.3 steel was carried out using scanning electron microscopy (SEM) on the samples near the lower shelf of the transition curve. The observation of SA508 Gr.4N steel was carried out using the Cv impact specimens at −130 °C, while the SA508 Gr.3 steel was carried on the impact specimens at −80 °C. The microscopic fracture in Figure 6 showed that the fracture surface was composed of a series of small cleavage planes, the river pattern morphology was formed on the cleavage steps, and the fracture surface was characterized by cleavage fracture. Most of the tear edges were obvious and smooth, and many slender river-like lines were distributed in each area, and the flow of the river was the direction of crack propagation. In the crack propagation zone, there were small areas of shallow dimpling (indicated by the red arrow in Figure 6), showing that there was still a certain ductile fracture zone at the tear end even in the brittle state. Secondary cracks existed in the instantaneous fracture zone of SA508 Gr.3 steel, and the cracks are relatively deep. Comparing the fracture characteristics of the two materials in Figure 6, it can be known that there was no significant difference in the river length between the two materials, but the cleavage step density of SA508 Gr.4N steel was slightly higher than that of SA508 Gr.3 steel. The distribution area of shallow dimples in SA508 Gr.4N steel was still larger than SA508 Gr.3 steel. Therefore, to a certain extent, it indicated that the fracture toughness of SA508 Gr.4N steel was superior.

**Figure 7:**
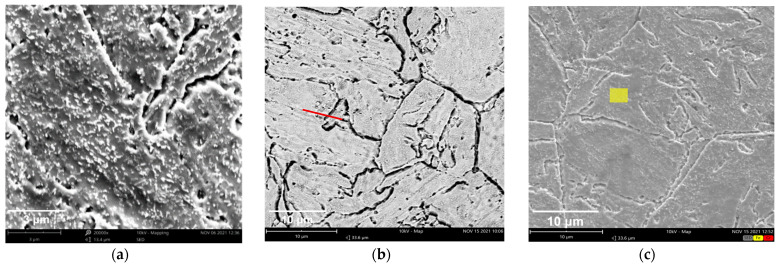
SEM images of metallographic specimen of SA508 Gr.4N steel. The white grain-liked dots in (**a**) represent carbides, the red line in (**b**) represents the scan line cross the grain boundary, and the yellow area in (**c**) represents the scan area within the grain.

SEM characterization of the SA508 Gr.4N metallographic specimen was carried out, and the results are shown in Figure 7. We can observe a large number of carbide particles, which were distributed on the surface of matrix structure of SA508 Gr.4N steel, and the distribution density was consistent with the reference [9]. The long diameter direction of the carbide was along the direction of the lath structure inside the grain. Since the internal lath of SA508 Gr.4N steel is finer and the Cr content is higher, the number of precipitates within the grains is greater and their size is smaller with an exact value of 0.05 μm~0.1 μm.

In order to prove the above analysis, the composition analysis of the X-ray energy spectrometer (EDS) in the inner region of the grain and the region across the grain boundary was carried out. The results are shown in Table 4. It was found that the Fe content was the highest, and the Cr, O and C were contained in a small amount, both in the ferrite grain and near the grain boundary. When the EDS line scanned through the grain boundary region, there was a sharp increase in C content or C and Cr content on the line sweep path, indicating that there was an obvious carbide precipitate on this path. So, it is likely to be a Cr-rich carbide precipitate (M7C3 or M23C6). This result is consistent with the reference [20]. At the same time, the probability of finding Cr element in the surface area of SA508 Gr.4N steel is higher than that of the SA508 Gr.3 steel examined in reference [20], which is consistent with the SEM observation.

#### 3.4.3. Electron Backscattering Diffraction Analysis

**Figure 8:**
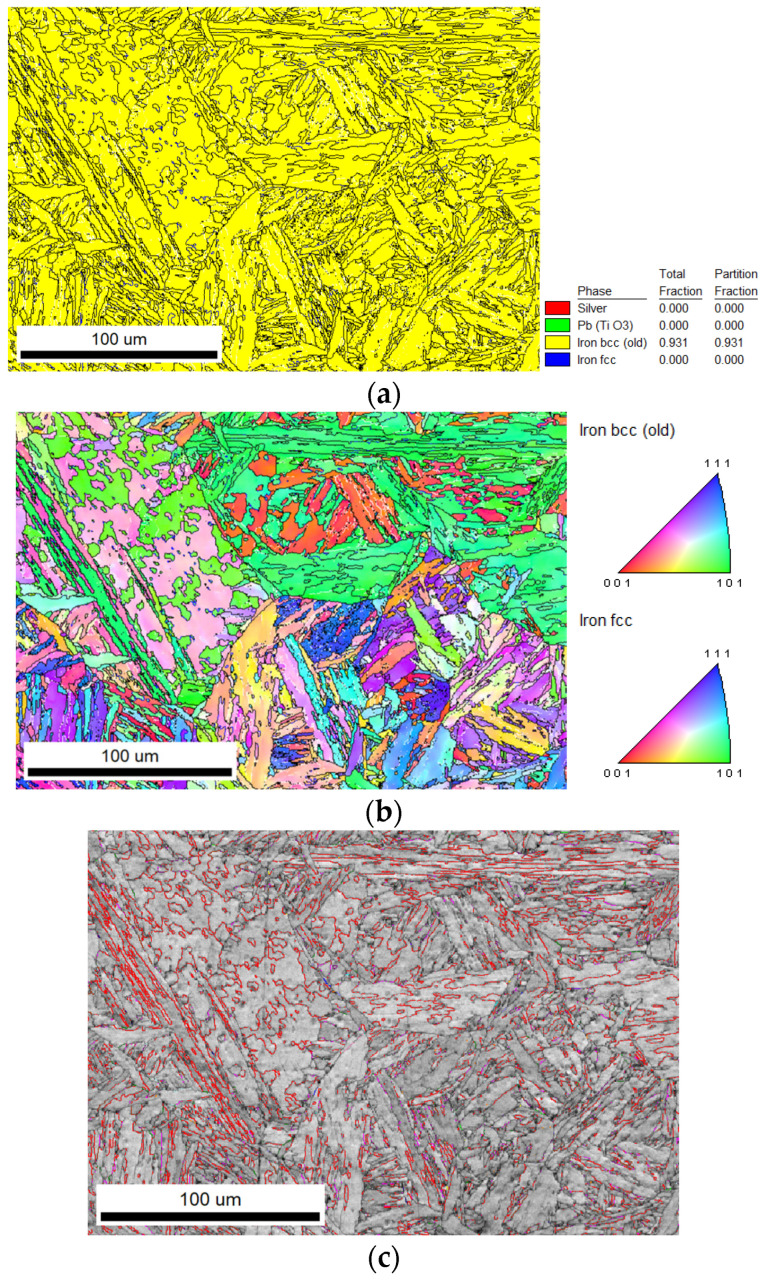
The EBSD of SA508 Gr.4N steel: (**a**) phase composition; (**b**) grain orientation; (**c**) grain boundary orientation.

Due to the limitation of samples, SA508 Gr.3 steel samples were not included in electron backscattering diffraction (EBSD) analysis, and only SA508 Gr.4N steel samples were included in the analysis results. The results of SA508 Gr.4N steel were compared with the results in reference [21]. The phase composition, grain orientation, grain boundary characteristics, and coincident site lattice (CSL) interface distribution of the material can be obtained from the EBSD analysis, and the test results are shown in Figure 8 below.

Figure 8a shows that the primary phase in the SA508 Gr.4N steel is the ferrite phase with a bcc structure, accounting for about 93% of the volume fraction. Figure 8b presents a grain orientation diagram in which different colors represent different distribution orientations. Orientations (001), (111) and (101) are shown in red, blue and green, respectively. It can be seen from Figure 8b that the morphology of lath blocks with different orientations mainly has two forms: one is a parallel distribution while the other is an interlaced distribution. The lath blocks with a parallel distribution have lower resistance to crack propagation, while the lath blocks with an interwoven distribution are not only beneficial to the suppression of crack propagation but are also beneficial to microstructure refinement and improve the impact toughness of the material. Figure 8c shows the grain boundary diagram. During data processing, the orientation difference of 15° ~ 100° is defined as the large-angle grain boundary represented by the black line, and an orientation difference of less than 15° is the small-angle grain boundary represented by the white line. It can be seen from Figure 8c that the proportion of large-angle grain boundary in the range of 15–100° is 35.7% and that of small-angle grain boundary is 12.8%. Figure 8c shows the CSL grain boundary diagram (red line), in which the proportion of ∑3 grain boundary is about 20.9%.

## 4. Discussion

### 4.1. Effect of Metallographic Structure on Fracture Toughness

Studies have shown that effective grain size is the key factor determining material toughness in low-alloy steels. Lath block (needle-like) is the smallest substructure determining toughness in SA508 Gr.4N steel and SA508 Gr.3 steel, i.e., the size of the lath block is an effective grain size that affects the low-temperature toughness of the materials [21]. In the structure of SA508 Gr.4N steel, the size of martensite lath is only half of bainite lath, produces more packet boundaries. The dense packet boundaries hinder the dislocation slip, which is macroscopically represented by the increase in tensile strength and fracture strength of the material.

The reason why the toughness of the martensite structure is higher than that of bainite is that the process of obtaining martensite requires a faster quenching cooling rate. A higher cooling rate weakens variant selection during the phase transition, which leads to the refinement of the lath blocks and an increase in large-angle grain boundaries. This is also the reason for the higher toughness of SA508 Gr.4N steel.

### 4.2. Effect of Precipitation on Relative Fracture Toughness

There were different sizes and distributions of carbide precipitates between SA508 Gr.4N steel and SA508 Gr.3 steel. The differences in their morphologies may lead to different properties. Yang’s research showed [22] that there was a linear relationship between impact energy and carbide size in low-alloy steel: as carbide size increases, impact energy decreases. Analysis of the reference temperature T_0_ of SA508 Gr.3 steel in references [23,24] found that the value of T_0_ was linearly related to the size of the critical carbide at the initiation of cleavage. The cleavage fracture was controlled by the initiation of microcracks in the carbide. The small-sized carbide with dispersion distribution was helpful in improving the cleavage fracture toughness in the transition zone [24]. Therefore, it can be speculated that when the crack is initiated in the material, the carbides will hinder its normal extension since the carbides are harder than the matrix. Then, the extension of the crack may be deflected. The deflection of the crack can increase the extension energy, thereby achieving a higher fracture toughness. So, the higher density distribution of the carbide, the stronger its ability to hinder crack propagation, and the higher fracture toughness of the materials are.

Also, a study by Lee et al. [25] on the critical crack size of SA508 Gr.3 steel showed that the critical crack size decreased with the decrease in fracture temperature. The critical crack size for a fracture at −75 °C was 0.414 μm, while it decreased to 0.151 μm when a fracture occurred at −196 °C. Therefore, reducing the size of the carbide precipitates to lower than the threshold for critical crack formation can effectively improve the low-temperature toughness, such that the reference temperature T_0_ of SA508 Gr.4N steel is lower than that of SA508 Gr.3 steel.

### 4.3. Effect of Grain Boundary Structure on Fracture Toughness

The lath morphology, grain orientation, and CSL boundaries of the two steels were analyzed using the EBSD technique, as shown in Figure 7. The average thickness of the martensite band is approximately 0.15 μm. Its proportion in volume is about 25%. The packet boundaries of martensite are basically CSL grain boundaries, which is consistent with the proportion of CSL ∑3 grain boundaries. According to the Hall–Petch relationship, the fine lath boundaries are similar to grain boundaries and can simultaneously enhance the strength and toughness of the material. The finer the grains are, the more interfacial resistance needs to be overcome for crack propagation.

The parallel distribution of the lath had lower resistance to crack propagation, and the interleaved distribution of the lath was beneficial to the suppression of crack propagation. The interleaved lath was also beneficial to the refinement of the tissue and the improvement of the impact toughness of the material. Because of the simple dislocation structure and the low grain boundary energy, the crack easily propagates. However, although large-angle grain boundaries can hinder crack propagation, due to the high grain boundary energy, it is also easy to cause impurity elements segregated in grain boundaries, leading to grain boundary weakening. The CSL grain boundaries have moderate grain boundary energy, which can hinder crack propagation but also cannot attract the segregation of solute atoms [26]. Studies have shown that the ∑3 grain boundary in nano-copper alloys is a low-energy state of a coherent twin grain boundary, which can not only hinder the movement of dislocations but can also absorb and store dislocations in motion as a slip surface when dislocations slip [27,28]. However, CSL grain boundaries are almost not present in the bainite plate of SA508 Gr.3 steel [12], which may also be the reason for the improvement of plastic toughness of SA508 Gr.4N steel.

## 5. Conclusions

The mechanical properties of SA508 Gr.4N steel and SA508 Gr.3 steel, especially the difference in fracture toughness, were studied in this article from the aspects of fracture characteristics, microstructure composition and second phase precipitation. The main conclusions are as follows:

1. Compared with SA508 Gr.3 steel, SA508 Gr.4N steel showed much better mechanical properties than SA508 Gr.3 steel in terms of tensile strength, impact energy and fracture toughness. In the selection of RPV steel, SA508 Gr.4N steel may be the optimal choice in terms of strength and toughness.
2. The size of martensite lath in SA508 Gr.4N steel is smaller than that of bainite in SA508 Gr.3 steel, which can create more sub-structural interfaces. Moreover, the CSL grain boundaries in SA508 Gr.4N steel have moderate grain boundary energy, which can not only block the crack propagation but also does not attract the segregation of impurity elements. It can improve the fracture toughness of the material.
3. The fine and diffuse carbide precipitates in SA508 Gr.4N steel can inhibit the crack propagation and dislocation slip. These precipitates make an important contribution to improving the cleavage fracture toughness and low-temperature toughness in the transition zone.

Irradiation embrittlement is a factor that must be considered for SA508 Gr.4N steel as an NPP material. The influence of its microstructure on fracture toughness under irradiation conditions needs to be further demonstrated, which will be the main focus of subsequent research.

## Figures and Tables

**Table 1:** Heat analysis of the chemical composition of SA508 steels.

Element	C	Si	Mn	P	S	Ni	Mo	Cr	V	Cu
SA508 Gr.4N	0.18	0.26	0.37	0.002	0.0002	3.46	0.54	1.76	0.006	0.02
SA508 Gr.3	0.19	0.18	1.44	0.005	0.002	0.74	0.51	0.14	0.005	0.02

**Table 2:** Tensile test values of SA508 Gr.4N steel and SA508 Gr.3 steel.

Designation	Temp (°C)	Yield Strength (MPa)	Tensile Strength (MPa)	Elongation (%)	Reduction (%)
SA508 Gr.4N	Room	666	786	26	75
SA508 Gr.3		431	568	29	76.5
SA508 Gr.4N	290	548	719	25	72
SA508 Gr.3		379	540	28	78

**Table 3:** Impact fracture characteristic values of SA508 Gr.4N steel and SA508 Gr.3 steel.

Designation	T_41J_ (°C)	T_68J_ (°C)	USE (J)	RT_NDT_ (°C)
SA508 Gr.4N	−130	−111.7	222.8	−120
SA508 Gr.3	−60	−50.5	198.5	−32

**Table 4:** Atomic weight content of EDS on the SA508 Gr.4N specimen in Figure 7b,c.

Element Symbol	Area (b)	Area (c)
Fe	89.34	97.20
O	5.94	/
Cr	2.92	2.80
C	1.80	/

## Data Availability

The original contributions presented in this study are included in the article. Further inquiries can be directed to the corresponding author.
